# Risk factors for low oocyte retrieval in patients with polycystic ovarian syndrome undergoing in vitro fertilization

**DOI:** 10.1186/s12958-023-01118-1

**Published:** 2023-07-19

**Authors:** Hao Jin, Haiyan Yang, Jiujia Zheng, Jiechun Zhou, Rong Yu

**Affiliations:** 1 The Urological Surgical Department, The First Affiliated Hospital of Wenzhou Medical University, No. 96, Fuxue Road, Lucheng District, Wenzhou, China; 2 The Reproductive Center, the First Affiliated Hospital of Wenzhou Medical University, Wenzhou, China

**Keywords:** Oocyte, Polycystic ovary syndrome, In vitro fertilization, Risk factor

## Abstract

**Background:**

The number of oocytes retrieved does not always coincide with the number of follicles aspirated in in vitro fertilization/intracytoplasmic sperm injection (IVF/ICSI) treatment. Patients with high expectation of retrieval sometimes obtain few oocytes, which may be induced by improper operation or therapeutic factors. The purpose of this study was to evaluate the distribution data of oocyte retrieval rate (ORR) and to explore the risk factors for low ORR in patients with polycystic ovary syndrome (PCOS) undergoing IVF/ICSI.

**Methods:**

A total of 2478 patients with PCOS undergoing IVF/ICSI were involved in this retrospective case-control study from March 2016 to October 2021. The oocyte retrieval rate was calculated as the ratio of the number of obtained oocytes to the number of follicles (≥ 12 mm) on the trigger day. Patients were divided into a low ORR and a normal ORR group with the boundary of one standard deviation from the mean value of ORR. The patient characteristics, treatment protocols, serum hormone levels, and embryonic and pregnancy outcomes were analyzed.

**Results:**

The ORR exhibited a non-normal distribution, with a median of 0.818. The incidence of complete empty follicle syndrome was 0.12% (3/2478). The proportion of patients in the low ORR group who received the progestin-primed protocol was significantly higher than that in the normal ORR group (30.30% vs. 17.69%). A logistic regression analysis showed that the serum estradiol level/follicle (≥ 12 mm) ratio (OR: 0.600 (0.545–0.661)) and progesterone level (OR: 0.783 (0.720–0.853)) on the trigger day were significant factors in the development of a low ORR, with optimal cutoff values of 172.85 pg/ml and 0.83 ng/ml, respectively, as determined by receiver operating curve. Fewer high-quality embryos (2 vs. 5) and more cycles with no available embryos (5.42% vs. 0.43%) were found in the low ORR group.

**Conclusions:**

For patients with PCOS, low estradiol levels/follicles (≥ 12 mm) and progesterone levels on the trigger day and the use of the progestin-primed protocol could be risk factors for low ORR, which leads to a limited number of embryos and more cycle cancellations.

## Background

Oocyte recovery is a key step in in vitro fertilization (IVF)/intracytoplasmic sperm injection (ICSI) cycles. A large number of studies focused on the ovarian response to gonadotropin stimulation [1, 2], but few have paid attention to the situation in which oocytes do not recover from a dominant follicle with normal ultrasound performance, known as an empty follicle [3]. The lack of oocytes obtained after all follicle puncturing is called empty follicle syndrome (EFS), an extreme condition of empty follicles that is very rare [4]. Because of operational contingency, not all patients who do not acquire oocytes are considered to suffer from EFS. In cycles with only 1–2 dominant follicles, failure to recover oocytes is not uncommon and to some extent acceptable [5]. However, patients with a high number of preovulation follicles sometimes also obtained oocytes that were much fewer than punctured follicles [6]. Although some of them were able to become pregnant [7], this kind of confusing outcome prompted us to consider whether it was due to an operator error or pathological oocyte development in the follicle.

According to Narguad’s research [8], the probability of expecting an oocyte from a mature follicle is 80%. This implies that the chance of obtaining no oocytes after puncturing 5–6 follicles in one patient should then theoretically be less than 0.01% [9]. As more follicles are punctured, the likelihood of failure or low oocyte retrieval should decrease with it, provided that the trigger medicine is correctly injected and occasional oocyte loss is permissible. Patients with polycystic ovary syndrome (PCOS) are suitable candidates for investigating cases of low oocyte retrieval because they have ample preovulation follicles and a high expected number of retrieved oocytes when the ovaries respond normally to gonadotropins. Different from the poor response to stimulation, empty follicle-related low oocyte retrieval suggests oogenesis abnormalities, such as zona pellucida packaging interruption, blocked cumulus complex detachment or dysfunctional granular cells in the process of oocyte formation and maturation [10–13].

The ratio of the number of oocytes obtained to the number of follicles punctured in IVF is generally believed to be close to 1, but the normal reference range has not been well specified, which is troubling in clinical practice to determine whether there is an abnormally low oocyte retrieval event. This study evaluated the oocyte retrieval rate (ORR) in a population of patients with PCOS undergoing IVF/ICSI to understand the routine distribution of this indicator. We also attempted to identify perceptible risk factors for low ORR, which could help to clarify the occurrence of such adverse events as an issue related to therapeutic options or patient characteristics, rather than a matter of operational accident.

## Methods

### Subjects

This retrospective case-control study sequentially collected 3056 fresh cycles of IVF or ICSI performed for patients with PCOS in the reproductive center of a single university-affiliated hospital from March 2016 to October 2021. The diagnosis of PCOS was in accordance with the Rotterdam conference standard [14]. The exclusion criteria were as follows: repeated cycles for the same patient, the discontinuation of ovarian stimulation, the cancellation of follicle puncture, cycles with serum luteinizing hormone (LH) level > 10 mIU/mL before the trigger day, and cycles with coasting. A total of 2478 patients were enrolled.

### Ovarian stimulation

At the time of inclusion, three stimulation protocols were used for patients with PCOS. Of all 2478 patients, 1256 underwent pituitary downregulation with a single injection of a gonadotrophin releasing hormone (GnRH) agonist (Triptorelin 3.75 mg, Ferring Pharmaceuticals, France), followed by stimulation with recombinant human follicle-stimulating hormone (r-FSH, Serono, Switzerland); 733 underwent the antagonist protocol, receiving 0.25 mg of GnRH antagonist (Cetrorelix Acetate, Serono, Switzerland) daily after 5–6 days of r-FSH administration; and 489 underwent the progestin-primed protocol, receiving 10 mg of medroxyprogesterone acetate (MPA) combined with human menopause gonadotrophin (HMG, Livzon Pharm, China) daily. The initial dose of r-FSH or HMG was 150–250 IU based on the patient’s body mass index (BMI) and age and was then adjusted according to the ovarian response estimated by trans-vaginal scans (Aloka Medical, Japan).

The trigger timing of the various protocols was consistent; that is, at least 2–3 follicles reached 18–20 mm in diameter. A total of 0.2 mg of GnRH agonist (Triptorelin 0.1 mg, Ferring Pharmaceuticals, France) or a combination with 1000–2000 IU of human chorionic gonadotropin (hCG, Livzon Pharm, China) was used in some of the antagonist and progestin-primed protocols. Beyond this, 250 µg of recombinant hCG (r-hCG, Serono, Switzerland) was administered for oocyte maturation in the remaining patients.

### Oocyte retrieval and embryo culture

The patients were asked to test their urine for hCG (or LH surge) at home on the day after the trigger to determine whether the solution was injected correctly. Patients with negative results were excluded from the study. Oocytes were retrieved 36–37 h after triggering under the guidance of transvaginal ultrasound. The follicles of bilateral ovaries with diameters of at least 12 mm were all punctured and aspirated using a 17G needle (COOK, USA) and under a pressure of 120 mmHg. During the period of this study, all punctures were performed by the same highly experienced physician. The follicular fluid was immediately checked by an experienced embryologist under a stereomicroscope (Olympus SZX10, Japan) at a magnification of 6–10 times. The number of oocytes with intact zona pellucida in the cumulus complexes (OCCs) was recorded.

OCCs were transferred to Vitrilife G-IVF™ (Vitrilife Sweden, Sweden) medium for further fertilization and culture following standard clinical practice [15]. Embryo quality grading was based on the standards described by Machtinger [16]. A high-quality embryo was defined as an embryo having at least six cells generated from cleavage in 3 days, with a generally uniform size and morphology and < 20% debris.

### Grouping

The oocyte retrieval rate (ORR) was defined as the ratio of the number of oocytes obtained at pick-up to the number of follicles visualized on ultrasound with a diameter ≥ 12 mm on the trigger day. Patients were divided into a low ORR group and a normal ORR group with the boundary of one standard deviation (one-sided) from the mean value of logarithmic transformed ORR. The basic characteristics, serum hormone levels, treatment data, and embryonic and pregnancy outcomes of the two groups were compared.

### Statistical analysis

The descriptive statistics and Shapiro‒Wilk test for normal distribution were performed on ORR and quantitative variables. Logarithmic transformation was used for ORR to determine the value of (mean + SD) as the criterion of grouping. The non-normally distributed variables are described in terms of the median and interquartile range and were compared between two groups using the Mann‒Whitney U test. To compare categorical variables, the chi-squared test or Fisher’s exact test was performed. A multivariate logistic regression analysis was performed to identify the possible independent risk factors for low ORR using candidate variables screened by intergroup comparison and adjusted for age and BMI. The candidate variables associated with serum hormone levels were stratified by quintiles before regression analysis considering that they had little effect on the outcome with a 1 unit increase (e.g., estradiol level, progesterone level). Three different ovarian stimulation protocols were assigned values of 1–3 according to the incidence of low ORR cases in each of them (antagonist protocol = 1, agonist protocol = 2 and progestin primed protocol = 3). Receiver operating curve (ROC) analysis was used to assess the predictive accuracy of the quantitative variables with significant differences in the regression analysis. The optimal cutoff value was determined by the Youden Index (maximizes (sensitivity + specificity − 1)).

All statistical analyses were performed using Statistical Package for Social Sciences version 22.0 (SPSS, IBM Corp, New York, USA). P values of < 0.05 were considered statistically significant.

## Results

**Fig. 1:**
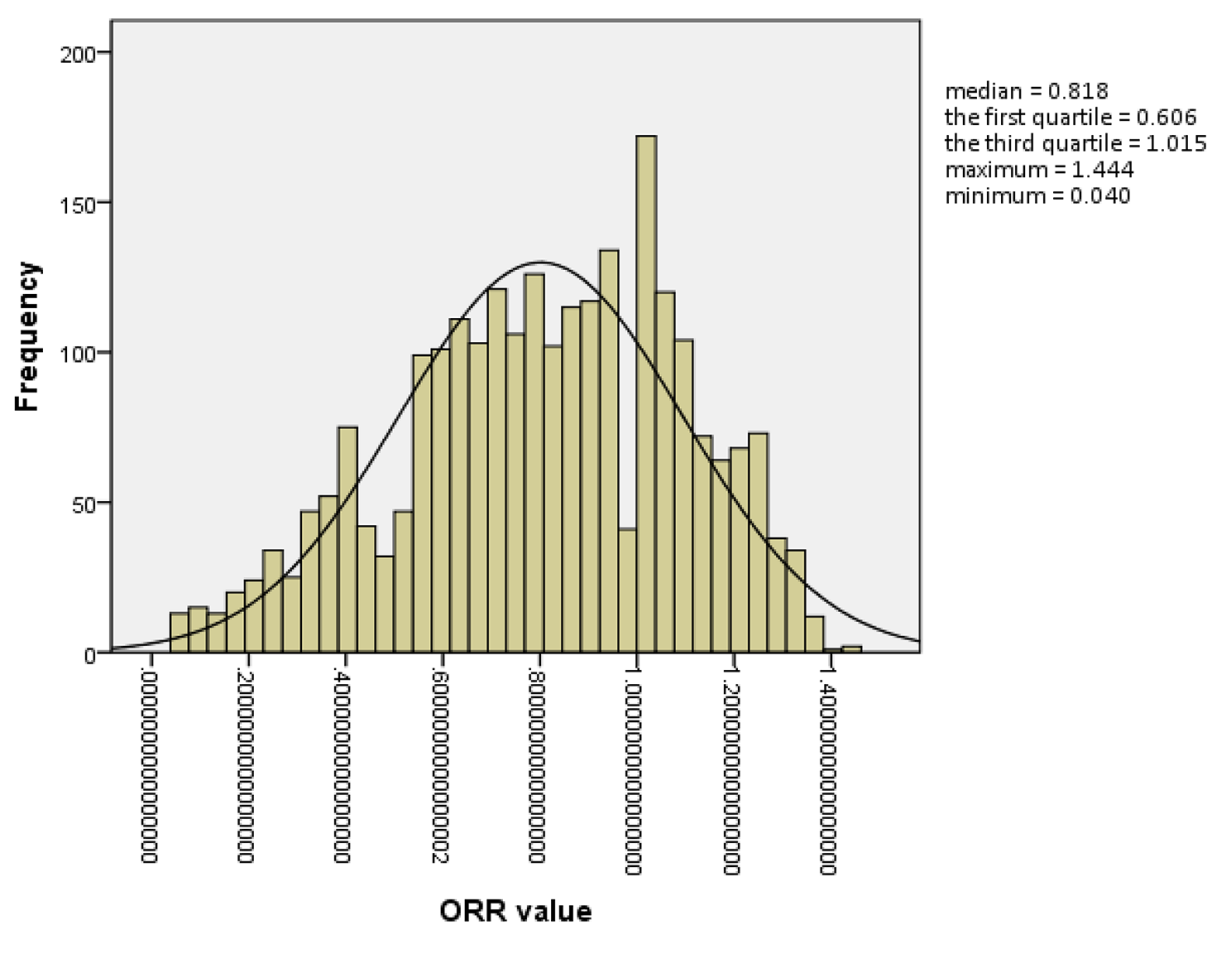
The non-normal distribution of the frequency of oocyte retrieval rate (ORR) values in patients with PCOS undergoing IVF/ICSI. ORR was defined as the ratio of the number of oocytes obtained to the number of follicles visualized on ultrasound with a diameter ≥ 12 mm on the trigger day

Cases of EFS with no oocytes obtained occurred in 3 (0.12%) of the 2478 included cycles. The characteristic and therapeutic data of these cases are described in detail in Table 1. In the 2475 cycles with at least one oocyte recovered, the distribution of the ORR was non-normal, as shown in Fig. 1, with a median of 0.818 and an interquartile range of 0.606–1.015, and the maximum and minimum values were 1.444 and 0.040, respectively. A normal distribution for the logarithmic (Ln)-transformed ORR was desirable, and the ORR value of 0.511 corresponding to the value of (mean + SD) of the transformed data was used as the criterion for grouping. Accordingly, 406 (16.40%) of all oocyte recovered cycles were assigned to the low ORR group (ORR < 0.511) and 2069 (83.60%) to the normal ORR group (ORR ≥ 0.511).

**Table 1:** Descriptive data of the clinical characteristics of patients with empty follicle syndrome

Characteristics	Patient 1	Patient 2	Patient 3
Age (y)	32.00	29.00	35.00
BMI (kg/m^2^)	24.97	19.20	23.61
Infertility years (y)	4.00	5.00	2.00
Infertility factors	primary/tubal factor/PCOS	primary/PCOS	primary/tubal factor/PCOS
Ovarian surgery history	none	none	none
Basal testosterone (ng/mL)	0.67	0.47	0.62
AMH (ng/mL)	8.06	7.67	6.38
Antral follicle count	37.00	32.00	40.00
Duration of stimulation (d)	14.00	10.00	12.00
Stimulation protocol	agonist downregulation	antagonist	antagonist
Trigger type	hCG	GnRH-a	hCG
*Hormones levels on the trigger day*			
E2 (pg/mL)	3849.86	5651.23	5363.22
LH (mIU/mL)	0.20	1.36	2.51
P (ng/mL)	1.11	1.48	1.33
E2/follicle (pg/mL)	213.88	226.05	268.16
Interval between trigger and aspiration (h)	36	37	36
No. of follicles ≥ 12 mm	18	25	20

Note: BMI, body mass index; PCOS, polycystic ovary syndrome; AMH, anti-Müllerian hormone; hCG, human chorionic gonadotropin; GnRH-a, gonadotrophin releasing hormone agonist; E2, estradiol; LH, luteinizing hormone; P, progesterone

Table 2 shows the comparison between the two groups. The results of the Mann–Whitney U test showed that when compared with patients in the normal ORR group, those in the low ORR group had more follicles (≥ 12 mm) visualized on ultrasound (25 (19-35) vs. 20 (15–26) , p < 0.001), a longer duration of stimulation (12 (10–15) vs. 11 (10-13), p < 0.001), lower serum progesterone levels (0.70 (0.48–1.04) ng/ml vs. 0.99 (0.67–1.37) ng/ml, p < 0.001), lower LH levels (1.57 (1.03–2.36) mIU/mL vs. 1.84 (1.21–2.89) mIU/mL, p < 0.001), lower estradiol (E2) levels/follicles (≥ 12 mm) on the trigger day (146.27 (103.36–186.75) pg/ml vs. 199.61 (151.38–253.25) pg/ml, p < 0.001) and fewer high-quality cleavage stage embryos (2 (1–4) vs. 5 (3–8), p < 0.001). In contrast, there was no significant difference in patient age, BMI, infertility years, basal testosterone or anti-mullerian hormone levels, the number of antral follicles, the E2 level on the trigger day, or the time interval between trigger and aspiration (p > 0.05). The results of the chi-square test showed that there was a significantly higher proportion of patients with primary infertility in the low ORR group than in the normal ORR group (56.90% vs. 51.14%, p = 0.034), but there was no difference in the proportion of various IVF indications, including tubal factors, endometriosis, and a history of ovarian surgery (p > 0.05). Regarding the clinical outcomes, the pregnancy rates per fresh embryo transfer were similar in both groups (56.65% vs. 62.20%, p > 0.05), but significantly more cycles with no available embryo (5.42% vs. 0.43%, p < 0.001) were found in the low ORR group. The incidence of severe ovarian hyperstimulation syndrome (OHSS) in the two ORR groups was statistically similar (p > 0.05).

**Table 2:** Comparison of patient characteristics and stimulation protocols between the low and normal ORR groups

Characteristics	ORR < 0.511 (n = 406)	ORR ≥ 0.511 (n = 2069)	P value
Age (y)	29.00 (27.00–32.00)	29.00 (27.00–32.00)	0.236
BMI (kg/m^2^)	23.48 (20.40–26.50)	22.89 (20.58–25.71)	0.140
Infertility years (y)	3.00 (2.00–5.00)	3.00 (2.00–5.00)	0.601
*Infertility factors*			
Primary infertility	231 (56.90)	1058 (51.14)	0.034
Tubal factor	209 (51.48)	1076 (52.01)	0.846
Endometriosis	11 (2.71)	36 (1.74)	0.191
History of ovarian surgery	4 (0.99)	13 (0.63)	0.505
Basal testosterone (ng/mL)	2.04 (1.52–2.34)	2.02 (1.53–2.21)	0.120
AMH (ng/mL)	6.55 (5.42–8.76)	6.50 (5.27–8.66)	0.285
Antral follicle count	30.00 (26.00–37.00)	30.00 (26.00–36.00)	0.193
Duration of stimulation (d)	12.00 (10.00–15.00)	11.00 (10.00–13.00)	< 0.001
*Stimulation protocol*			
Agonist downregulation	209 (51.48)	1046 (50.56)	
Antagonist	74 (18.23)	657 (31.75)	
MPA	123 (30.30)	366 (17.69)	< 0.001*
*Trigger type*			
recombinant -hCG	295 (72.66)	1355 (65.49)	
GnRH-a	90 (22.17)	529 (25.57)	
GnRH-a + hCG	21 (5.17)	185 (8.94)	0.007 *
*Hormones levels on the trigger day*			
E2 (pg/mL)	3748.43 (2408.09-5250.37)	3937.33 (2646.87-5523.43)	0.059
LH (mIU/mL)	1.57 (1.03–2.36)	1.84 (1.21–2.89)	< 0.001
P (ng/mL)	0.70 (0.48–1.04)	0.99 (0.67–1.37)	< 0.001
E2/follicle (pg/mL)	146.27 (103.36-186.75)	199.61 (151.38-253.25)	< 0.001
Interval between trigger and aspiration (h)	36.00 (36.00–37.00)	36.00 (36.00–37.00)	0.593
No. of follicles ≥ 12 mm	25.00 (19.00–35.00)	20.00 (15.00–26.00)	< 0.001
No. of retrieved oocytes	8.00 (5.00–12.00)	17.00 (13.00–23.00)	< 0.001
No. of high-quality embryos	2.00 (1.00–4.00)	5.00 (3.00–8.00)	< 0.001
Cycles with no available embryos	22 (5.42)	9 (0.43)	< 0.001
Clinical pregnancy per fresh embryo transfer	115/203 (56.65)	436/701 (62.20)	0.154
Severe OHSS	7 (1.72)	28 (1.35)	0.563

Note: Data are presented as the median (interquartile range) or numbers (percentage). P values are from the Mann–Whitney U test, the χ2 test or Fisher’s exact test

* P values of the χ^2^ test among the three treatment groups

ORR, oocyte retrieval rate; BMI, body mass index; AMH, anti-Müllerian hormone; MPA, medroxyprogesterone acetate; hCG, human chorionic gonadotropin; GnRH-a, gonadotrophin releasing hormone agonist; E2, estradiol; LH, luteinizing hormone; P, progesterone; OHSS, ovarian hyperstimulation syndrome

**Fig. 2:**
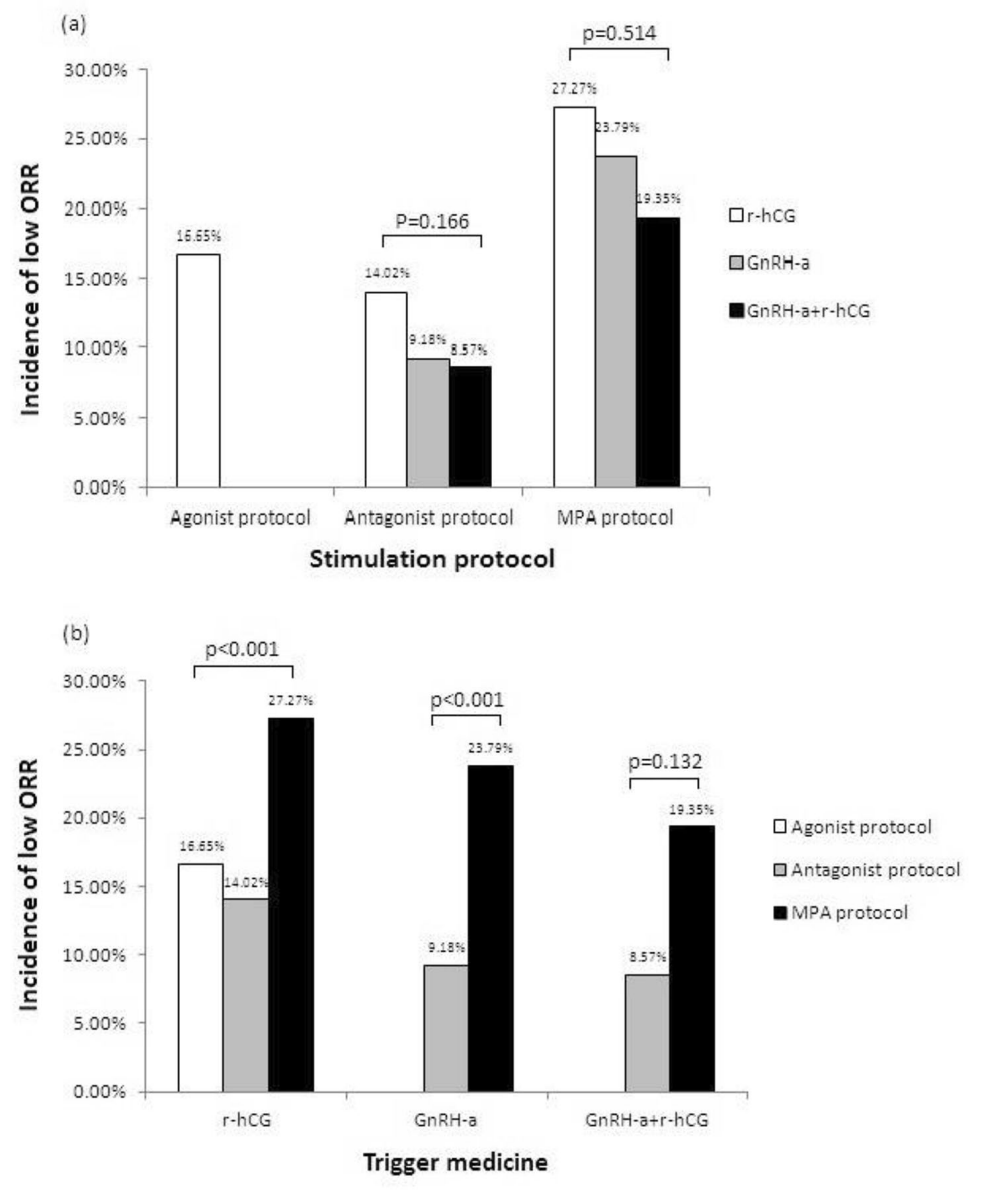
(**a**) Comparison of low ORR events occurring in conditions of different triggers with the same ovarian stimulation protocol by the χ2 test or Fisher’s exact test. (**b**) Comparison of low ORR events occurring in conditions of different ovarian stimulation protocols with the same trigger administration by the χ2 test or Fisher’s exact test MPA, medroxyprogesterone acetate; r-hCG, recombinant human chorionic gonadotropin; GnRH-a, gonadotrophin releasing hormone agonist

The proportions of stimulation protocols and trigger medicines used showed significant differences between the two groups (p < 0.001). Considering the impact of different combinations of ovarian stimulation and trigger strategies on the ORR [17], a post hoc analysis was conducted, as shown in Fig. 2. There was no difference in the incidence of low ORR events among patients undergoing each identical ovarian stimulation protocol, regardless of which trigger medicine was used (P > 0.05). In contrast, when r-hCG and GnRH agonist were used as triggers, the patients undergoing the progestin-primed protocol had a significantly higher incidence of low ORR events than those undergoing other stimulation protocols (P < 0.001), whereas the difference became nonsignificant when dual hCG and GnRH agonist triggering was used (P = 0.132).

**Fig. 3:**
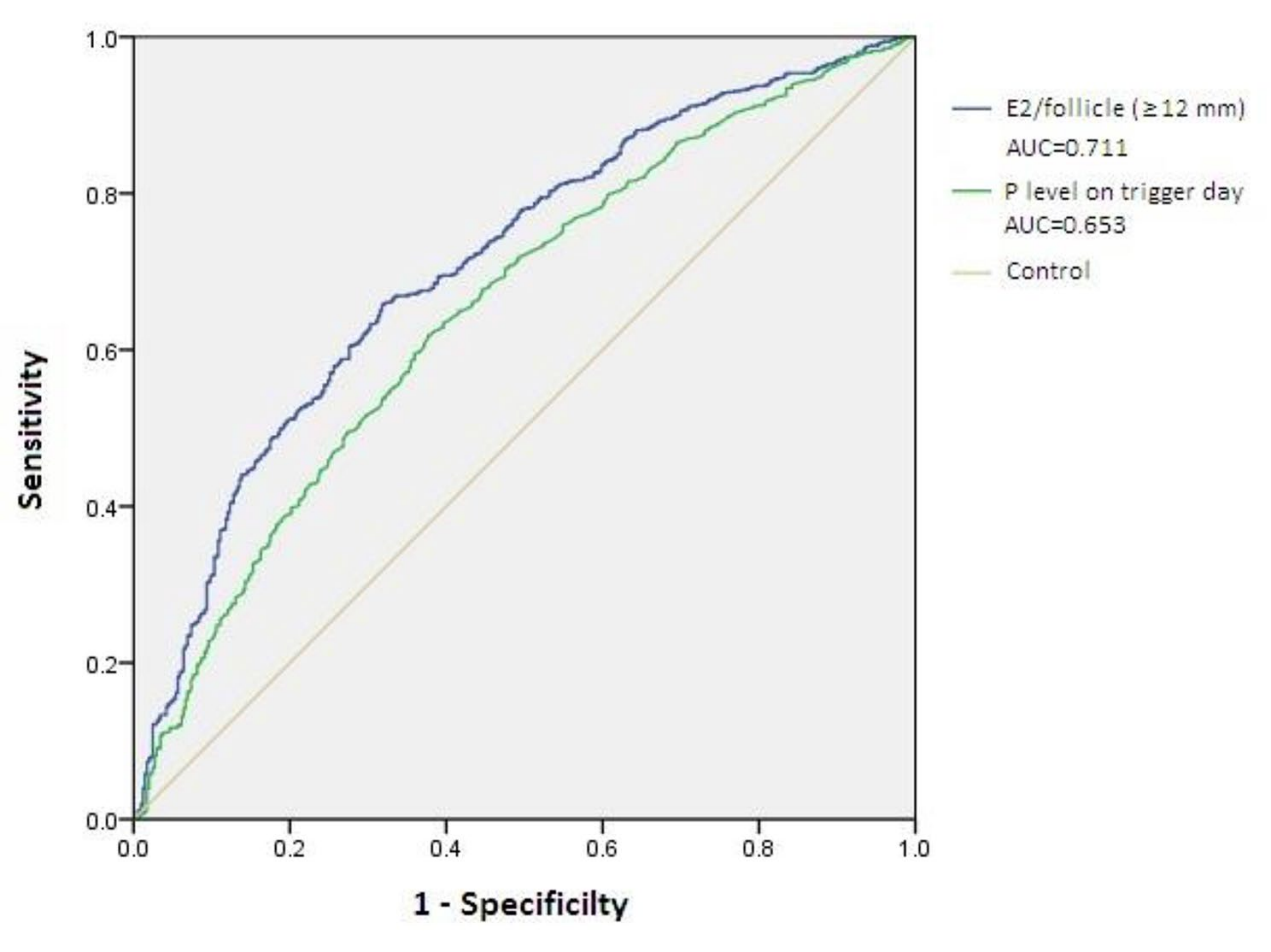
Receiver operating curve analyses of the ratio of the estradiol (E2)/follicle (≥ 12 mm) and progesterone (P) level on the trigger day to predicting low ORR events

The results of multivariate logistic regression analysis demonstrated that when adjusted for patient age and BMI, the adjusted ORs (95% CI) of the serum E2/follicle (≥ 12 mm) ratio, progesterone level on the trigger day and various stimulation protocols for low ORR events were 0.600 (0.545–0.661) (p < 0.001), 0.783 (0.720–0.853) (p < 0.001) and 1.596 (1.342–1.897) (p < 0.001), respectively. Further ROC analyses, presented in Fig. 3, revealed that the serum E2 level/follicle (≥ 12 mm) ratio and progesterone level on the trigger day had AUC values of 0.711 and 0.653, with cutoff values of 172.85 pg/ml and 0.83 ng/ml, respectively, for predicting low ORR events.

## Discussion

There were two forms of low ORR cases observed in this study, one in which oocytes were not retrieved at all, that is, meeting the criteria for EFS, and the other in which few oocytes were retrieved. The prevalence of genuine EFS presently remains unclear [18]. Here, we reported an incidence of EFS of 0.12% (3/2478) in patients with PCOS, which is consistent with the assertion of the rarity of this disease [4]. Because of the small number of cases, no statistical analysis was performed; however, no similarities in the clinical parameters were observed among these patients. Specifically, the treatment protocols of the three patients were different, and they had no risk factors, such as a low E2/follicle (≥ 12 mm) ratio or low progesterone level, that were present in patients with a low ORR who had retrieved oocytes. The EFS-associated underlying mechanism was speculated to be genetic changes (LHCGR, ZP1/2/3, etc.) as previously reported [10, 11] rather than therapeutic factors. Notably, two of these three patients underwent the second treatment of ovarian stimulation and oocyte retrieval 4 and 10 months after this failure, respectively, and still did not obtain an oocyte.

The non-normal distribution of ORR values was as expected, clustered in the range of 0.8-1.0, which corresponded to the operational loss rate predicted by Nargue et al. [8]. Although the diameter of target follicles to be punctured according to our Standard Operating Procedure is at least 12 mm, the diameter is visually measured during the puncturing. Moreover, the number of dominant follicles in patients with PCOS is relatively large, and there may be a subtle error between it and the record count. These probably lead to the aspiration of more follicles than recorded and an ORR greater than 1 in some cases. Due to the lack of accepted criteria, we formulated a low oocyte retrieval situation with a log-transformed value of ORR beyond 1 standard deviation of the mean (one-sided) of the observed population in this study. Thus, an ORR with a value nearly below 0.5 was suggested to be taken seriously by physicians. This made it possible to distinguish and survey low ORR events from IVF treatments, although the criterion was exploratory and may be more accurate in the future.

There are multiple options of ovarian stimulation for patients with PCOS undergoing IVF [19]. Among these methods, the antagonist protocol, as an alternative to classic pituitary downregulation, is thought to achieve oocyte retrieval and pregnancy rates that are comparable to those of the agonist protocol [20, 21]. In contrast, the therapeutic outcomes of the progestin-primed protocol, in which MPA is used instead of the GnRH analog, are controversial [22–24]. Our study showed that a significantly higher proportion of MPA was used in the low ORR group, suggesting that this protocol would negatively affect the ORR in patients with PCOS. The severe LH suppression induced by MPA was considered to be the cause of the decreased oocyte number in a previous study [25], and the dual trigger of hCG combined with GnRH agonist has been shown to improve the ORR [26]. From the post hoc analysis of different combinations of ovarian stimulation and trigger administration, we further demonstrated that dual triggering, as a strategy to improve the ORR, could be advisable in the progestin-primed protocol but is not necessary in antagonist cycles.

Another predictor of low ORR found in the study was the ratio of serum E2 level to the number of follicles (≥ 12 mm) visualized on ultrasound on the trigger day. The serum level of E2 secreted by proliferative granulosa cells is commonly used as a hormonal marker of ovarian response for stimulation [27]. It has been proven to be positively correlated with the number of retrieved oocytes and to have a subsequent impact on the pregnancy outcome [28, 29]. Especially in cycles with few follicles, the peak E2 level is considered a predictor of oocyte maturation [30]. However, the exact E2 concentration for identifying the number or maturation rate of oocytes has not been established [31, 32], because the E2 level is strongly influenced by the number of preovulatory follicles, which varies considerably among patients even with the similar ovarian reserve. As in our study, the difference of peak E2 level between the two groups was not significant, and the number of follicles (with diameter ≥ 12 mm) visualized on ultrasound also did not show a significant effect in the regression analysis, suggesting that neither of them could be used to predict low ORR.

The peak E2 level instead of the E2/follicle ratio is convenient to use but is not suitable for patients with a number of follicles that is far beyond that of the general population. A decrease in the E2/follicle ratio might be seen when patients with PCOS have peak E2 levels that are parallel to those of other patients, and this hides the risk of oocyte immaturity and a low ORR. Moreover, the large numbers of predominant follicles contribute to the production of serum E2, leading to a lower average E2 level of each follicle, which is also responsible for the development of a low ORR event. Therefore, the use of the E2/follicle ratio is preferred over the peak E2 level as the hormonal marker to estimate the oocyte maturity of patients with PCOS, and a certain concentration, such as the concentration of 172.85 pg/ml resulting from the ROC analysis of this study, could be determined by individual clinical units. Admittedly, the early administration of the trigger would sometimes be used to prevent OHSS even if the E2/follicle ratio is insufficient [33], and physicians should be aware of the risk of a low ORR in this condition.

In our study, the progesterone level on the trigger day showed statistical significance in both the intergroup comparison and regression analysis; although the ROC analysis suggested that its predictive effect of a low ORR was not as strong as that of the E2/follicle ratio. Like estradiol, progesterone is a product secreted by ovarian granulosa cells, and the concentration of progesterone increases with the development of follicles and is positively associated with the number of oocytes [34–36]. It was found that both high and low progesterone levels in the late follicular phase resulted in reduced oocyte numbers [37]. High levels may indicate premature ovulation, while low levels suggest insufficient proliferation or impaired function of granulosa cells, implying that the follicle is not yet close to maturity. The results of our study are consistent with this conclusion.

To date, few studies have described the impact of empty follicle related low oocyte retrieval on pregnancy outcomes in IVF. In this research, we found that the clinical pregnancy rates per fresh transfer were similar in patients of different ORR groups, suggesting that empty follicle related low ORR did not affect the developmental potential of the embryos with the best quality, which are used for the first fresh transfers. However, as expected, the results demonstrated a significant decrease in the number of high-quality embryos, with an average of only 2, and a higher proportion of cycles with no available embryos, reaching 5.42% in the low ORR group. It can be speculated that a low ORR will give rise to no or little chance of transfer for many couples and a lower cumulative pregnancy rate per started cycle. Undoubtedly, the prediction of a low ORR will help to prevent these adverse outcomes.

## Conclusions

This retrospective observational study described the distribution of oocyte retrieval rates in patients with PCOS undergoing IVF/ICSI. It was identified that the E2 level/follicle (≥ 12 mm) ratio as well as the progesterone level on the trigger day and the use of the progestin-primed protocol were risk factors for low ORR, in which the predictive cutoff values of the E2/follicle ratio and progesterone levels were also established. We inferred a decreased cumulative pregnancy rate based on the reduced number of high-quality embryos but did not present definitive data due to the limited follow-up period. Whether patients with a low ORR suffer from genetic mutations similar to those with EFS and the available rescue treatment need to be further studied.

## Data Availability

The datasets analyzed during the current study are available from the corresponding author on reasonable request.

## Abbreviations

IVF: In vitro fertilization
ICSI: Intracytoplasmic sperm injection
EFS: Empty follicle syndrome
PCOS: Polycystic ovary syndrome
ORR: Oocyte retrieval rate
LH: Luteinizing hormone
GnRH: Gonadotrophin releasing hormone
FSH: Follicle stimulating hormone
MPA: Medroxyprogesterone acetate
HMG: Human menopause gonadotrophin
BMI: Body mass index
hCG: Human chorionic gonadotropin
ROC: Receiver operating curve
E2: Estradiol
